# Biomechanical comparison of high hydrostatic pressure versus liquid nitrogen treatment for devitalized bone grafts: a fresh porcine model study

**DOI:** 10.1186/s12891-026-09943-2

**Published:** 2026-05-08

**Authors:** Satoshi Nagatani, Satoshi Kato, Tetsuji Yamaoka, Shinji Miwa, Noriaki Yokogawa, Takaki Shimizu, Yohei Yamada, Michiharu Sakamoto, Eiichi Sawaragi, Rie Akita, Naoki Morimoto, Satoru Demura

**Affiliations:** 1 Department of Orthopaedic Surgery, Graduate School of Medical Sciences, Kanazawa University, 13-1 Takara-machi, Kanazawa, 920-8641 Ishikawa Japan; 2 Department of Clinical Engineering, Faculty of Health Sciences, Komatsu University, He 14-1, Mukai-motoori-machi, Komatsu, 923-0961 Ishikawa Japan; 3 Department of Plastic and Reconstructive Surgery, Graduate School of Medicine, Kyoto University, 54 Kawahara-cho, Shogoin, Sakyo-ku, Kyoto, 606-8507 Japan

**Keywords:** High hydrostatic pressure, Devitalized bone graft, Liquid nitrogen, Mechanical strength, Porcine model

## Abstract

**Background:**

Devitalized bone grafting is a widely used reconstructive technique after tumor resection. Conventional devitalization methods, such as autoclaving and irradiation, compromise the biological and mechanical properties of bone. Cryotreatment with liquid nitrogen has been associated with relatively early bone union; however, concerns remain regarding reduced initial mechanical strength and the risk of nonunion. High hydrostatic pressure (HHP) has been proposed as a novel devitalization method that may better preserve bone strength. This study aimed to compare the mechanical properties of fresh porcine bone treated with HHP versus liquid nitrogen.

**Methods:**

Fresh, unfrozen porcine vertebrae and femurs were collected and processed within 72 h to avoid confounding mechanical effects of prior freezing. Trabecular bone cubes (10 mm per side) from the vertebrae underwent compressive testing (*n* = 10 per group), and cortical bone beams (5 mm wide) from the femurs underwent three-point bending tests (*n* = 12 per group). Specimens were allocated to three groups: HHP-treated (200 MPa, 10 min), liquid nitrogen-treated (− 196 °C, 20 min), and untreated control. Mechanical testing was performed using a universal testing machine. One-way analysis of variance with Tukey’s post hoc test (*p* < 0.05) was used for statistical analysis.

**Results:**

In trabecular bone compression testing, both HHP-treated and liquid nitrogen-treated groups demonstrated lower maximum compressive load at failure than the control group. A significant difference was observed only between the liquid nitrogen-treated and control groups (*p* = 0.003). The difference between the HHP-treated and control groups was not significant (*p* = 0.07), and no significant difference was found between the two treatment groups (*p* = 0.39). In cortical bone three-point bending tests, no significant differences in maximum load at failure were observed among the groups (*p* = 0.37).

**Conclusions:**

HHP treatment did not significantly compromise mechanical properties compared with the untreated control, whereas liquid nitrogen treatment significantly reduced the maximum compressive load at failure of trabecular bone. These findings suggest that HHP may represent a structurally viable devitalization method for bone grafts, particularly in load-bearing applications.

## Background

Excisional surgery for bone tumors frequently creates substantial bone defects. Reconstruction typically necessitates bone grafting. Allogeneic bone grafting is commonly used to repair such defects. However, clinical complications such as nonunion, fracture, and infection have been associated with the use of allogeneic grafts [1]. Thus, devitalized autologous bone grafts are used as an alternative for reconstruction. Various methods have been developed for preparing devitalized bone grafts, including autoclaving and irradiation. Although these approaches are effective for sterilization, they are known to compromise the biological and mechanical properties of devitalized bone grafts [2–6]. Among the current methods, liquid nitrogen treatment has been reported to enable relatively early bone union [7, 8]. However, this technique has several limitations, including reduced osteogenic potential, impaired vascularization, and diminished initial mechanical strength [9].

High hydrostatic pressure (HHP) has emerged as a novel devitalization technique to overcome these challenges. HHP was initially developed for sterilization and preservation in the food industry [10–12]. It has since been applied in the medical field for uses such as sterilization and vaccine production [13–16]. HHP inactivates cells by applying isotropic pressure evenly throughout the tissue [17–19]. Impotantly, it preserves the structural and mechanical characteristics of the tissue [20, 21].

The preservation of mechanical strength is a key factor for the clinical success of devitalized bone grafts. In the early postoperative phase, the devitalized bone graft must endure mechanical loads to support patient movement and rehabilitation. Insufficient mechanical strength may result in complications such as fracture, deformity, or the need for revision surgery, all of which may negatively affect the outcomes. Despite increasing interest in HHP-treated bone, data on its mechanical properties remain limited. To address this gap, this study aimed to evaluate the mechanical strength of bone treated with HHP and to provide foundational data to support its clinical application as a structurally reliable devitalized bone graft.

## Methods

### Specimen collection and storage

Fresh porcine vertebrae and femurs were used in this study. All specimens were obtained from a local slaughterhouse (Tokyo Shibaura Organ Co. Ltd., Japan), stored at 4 °C, and processed within 72 h to maintain biological and structural integrity.

### Specimen preparation and group allocation

**Fig. 1:**
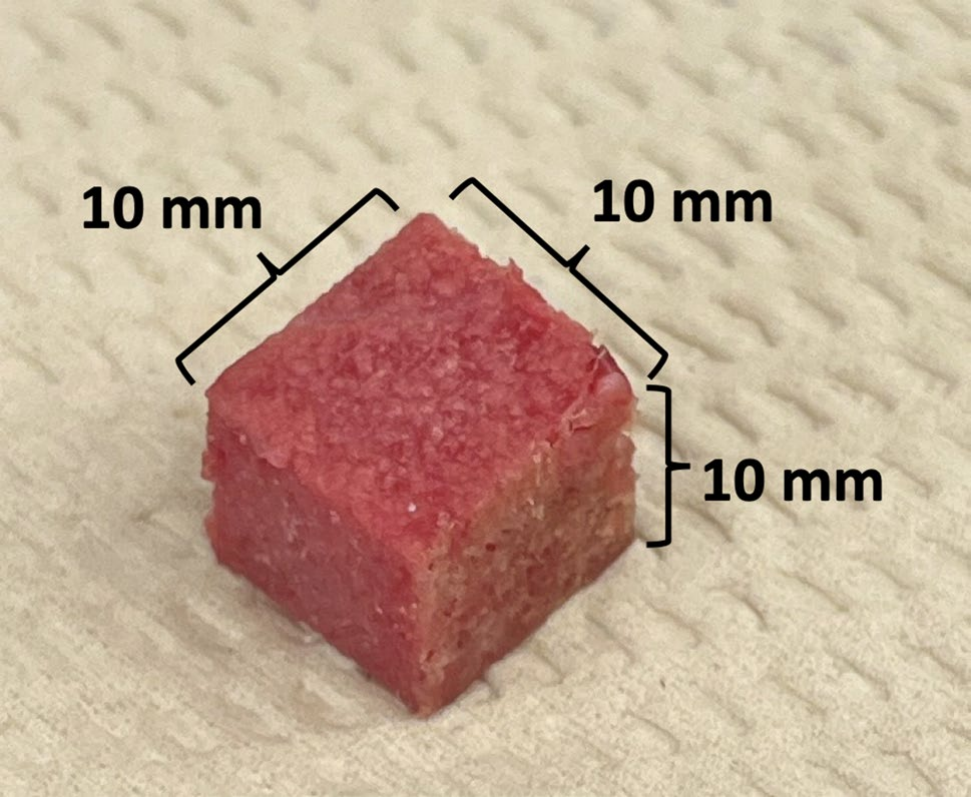
Representative image of a porcine trabecular bone specimen shaped into a 10 mm cube

**Fig. 2:**
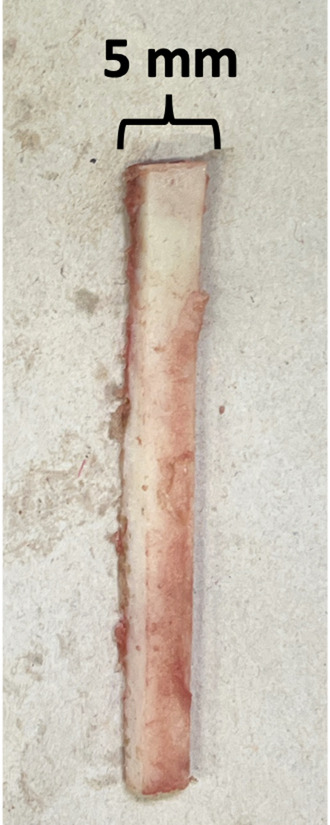
Representative image of a porcine cortical bone specimen shaped into a 5-mm-wide beam-shaped specimen

Trabecular bone was collected from lumbar vertebrae and shaped into cubes measuring 10 mm on each side (Fig. 1). Cortical bone was obtained from the anterior femur and processed into beam-shaped specimens with a width of 5 mm (Fig. 2). The prepared specimens were divided into three groups: HHP-treated, liquid nitrogen-treated, and untreated control. Specimens were allocated to the three groups during specimen preparation without selection based on specimen appearance or dimensions to minimize selection bias.

### Bone treatment protocols

**Fig. 3:**
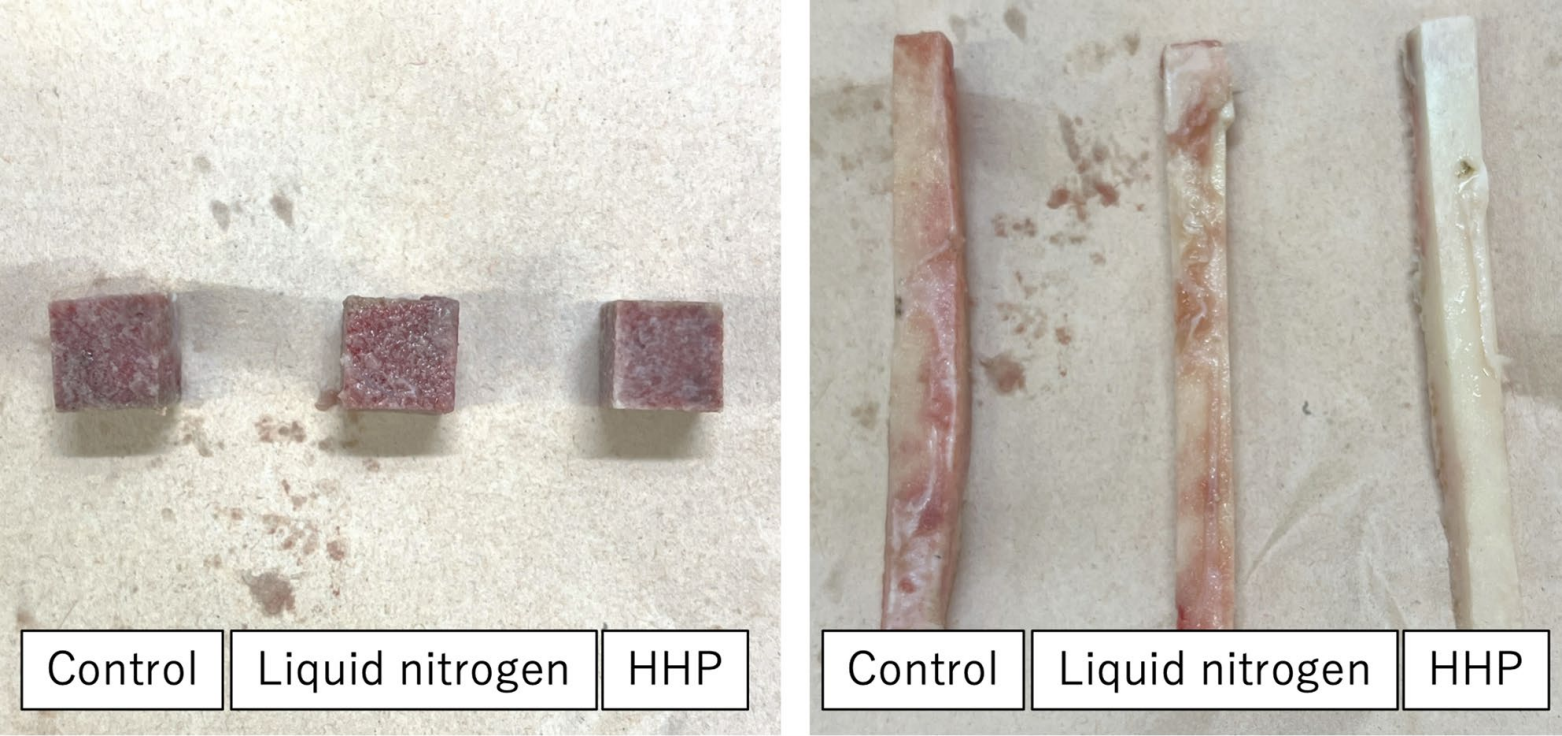
Macroscopic appearance of bone specimens following treatment. Legend: Representative images of trabecular bone cubes and beam-shaped cortical bone specimens. From left to right: untreated control, liquid nitrogen-treated, and HHP-treated groups. Note that no obvious gross discoloration or deformation was observed following either treatment

For HHP treatment, the specimens were sealed in sterile plastic bags filled with saline and placed in a chamber filled with tap water in an isotropic pressure device (ECHIGO SEIKA Co., Ltd., Nagaoka, Japan). A pressure of 200 MPa was applied for 10 min. This pressure and duration setting (200 MPa for 10 min) was selected based on previous reports that confirmed that this specific protocol is sufficient for tumor devitalization [22–26]. For liquid nitrogen treatment, the specimens were immersed in liquid nitrogen at − 196 °C for 20 min, then thawed for 15 min at room temperature, followed by 15 min in saline at ambient temperature. This protocol was adopted based on the standard clinical method established for tumor devitalization [27]. Both the cortical and trabecular bone specimens were processed using identical HHP or liquid nitrogen treatment protocols within the same experimental setup to ensure consistency across sample types. The control group specimens received no treatment. The appearance following each treatment is shown in Fig. 3.

### Mechanical testing

Mechanical testing was performed using a universal testing machine (EZ Test, Shimadzu, Japan). Prior to testing, the machine was calibrated according to the manufacturer’s specifications to ensure load measurement accuracy.

### Compressive testing of trabecular bone cubes

**Fig. 4:**
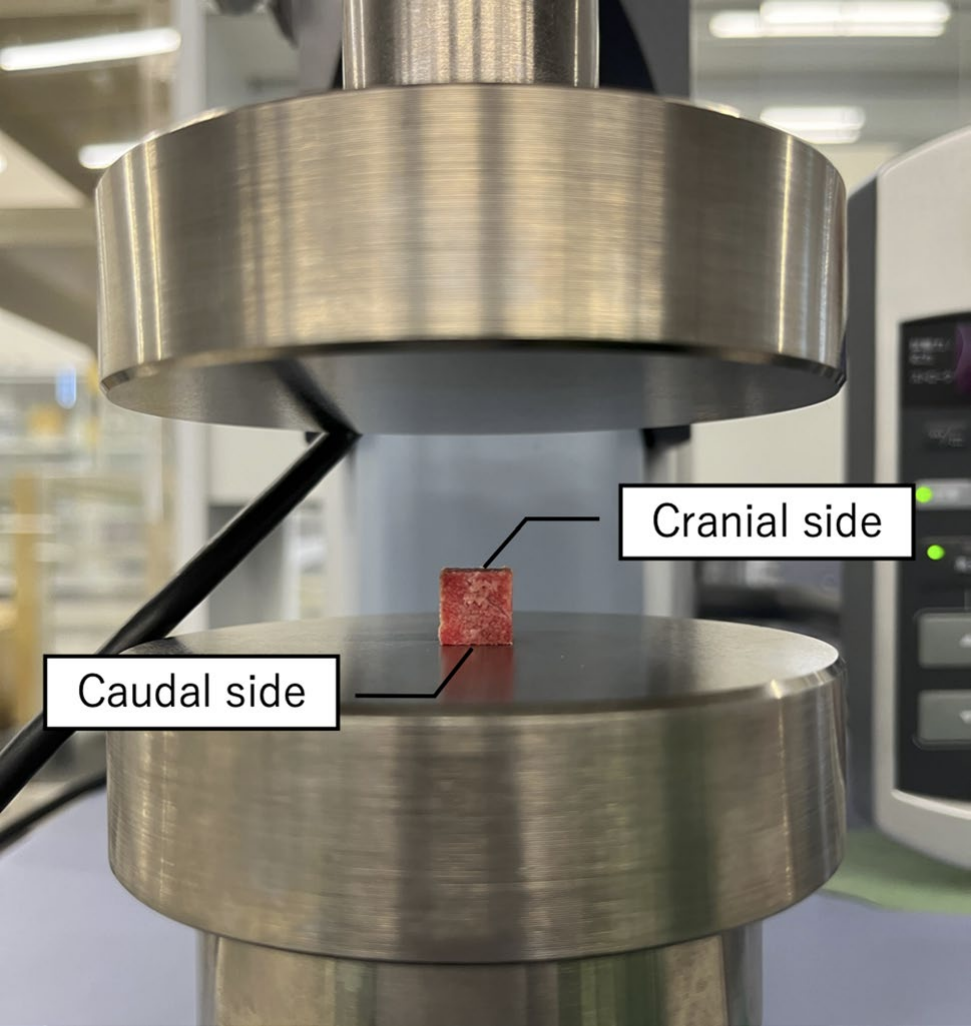
Experimental setup for compressive testing of trabecular bone. Legend: A trabecular bone cube was placed between the plates of a universal testing machine. A compressive load was applied vertically at a crosshead speed of 1 mm/min

A total of 10 trabecular bone cubes were prepared for each group. Compressive testing was conducted using a universal testing machine (EZ Test, Shimadzu, Japan). The specimens were placed in the testing machine with the anatomical cranial side facing upward and the caudal side facing downward (Fig. 4). A compressive load was applied vertically at a rate of 1 mm/min until structural failure. The maximum compressive load at failure (N) was recorded for each specimen and used for intergroup comparison.

### Three-point bending test of beam-shaped cortical bone

**Fig. 5:**
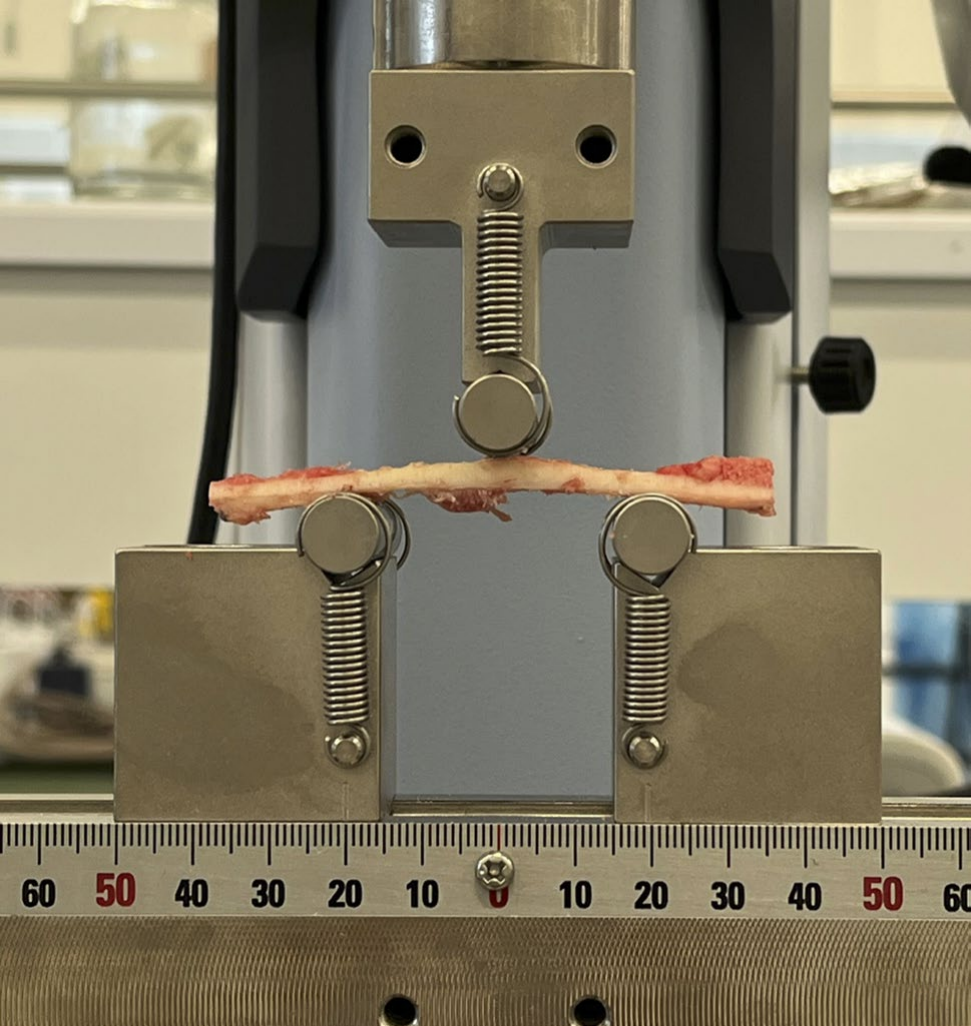
Experimental setup for three-point bending test of cortical bone. Legend: A cortical bone beam was mounted on a three-point bending fixture with a support span of 40 mm. A load was applied to the center of the span at a crosshead speed of 1 mm/min

A total of 12 beam-shaped cortical bone specimens were prepared for each group. Each specimen was mounted on a three-point bending fixture with the anterior (cortical) surface facing upward. The span between supports was set to 40 mm. A vertical load was applied at 1 mm/min until fracture (Fig. 5). Testing was conducted using the same universal testing machine. The maximum load at failure (N) was recorded for each specimen.

### Statistical analysis

The normality was evaluated using the Shapiro–Wilk test and the homogeneity of variances was evaluated using Levene’s test. After the assumptions were met, one-way analysis of variance (ANOVA) was performed, followed by Tukey’s post hoc test for multiple comparisons. A p-value < 0.05 was considered statistically significant. The sample size was determined based on previous studies that investigated bone biomechanics [27, 28]. However, as variance estimates specific to our specimen geometry and loading conditions were unavailable, an a priori power analysis was not performed. Instead, a sensitivity analysis indicated that the sample size of 10–12 specimens per group provided 80% power to detect large effect sizes (Cohen’s f ≈ 0.55–0.60) at a significance level of α = 0.05.

## Results

All values are expressed as mean ± standard deviation (SD).

### Compressive Test (Maximum Compressive Load at Failure)

**Fig. 6:**
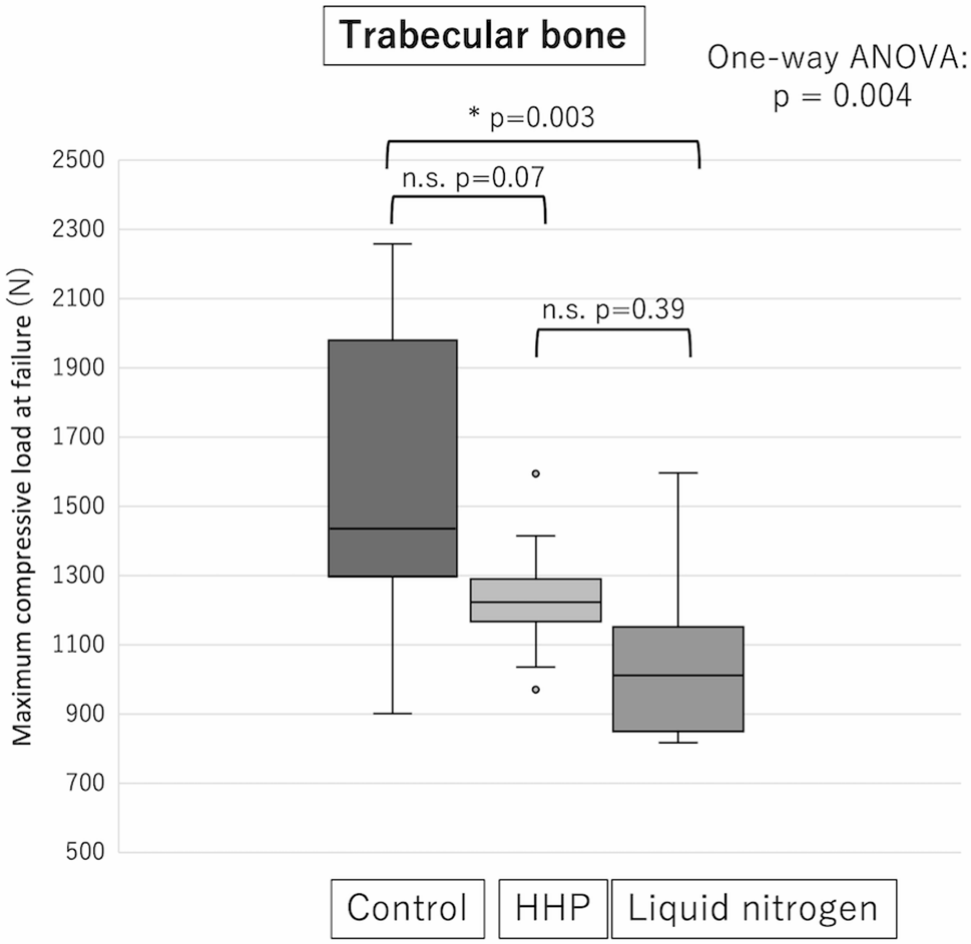
Maximum compressive load at failure of trabecular bone following HHP, liquid nitrogen, or no treatment. Legend: Box plots representing the median, interquartile range, and range of the maximum compressive load at failure for trabecular bone specimens (*n* = 10 per group). One-way ANOVA revealed a significant difference among the groups (*p* = 0.004). Post hoc analysis showed that the liquid nitrogen-treated group had a significantly lower maximum compressive load at failure than the control group (*p* = 0.003; Tukey’s test). The difference between the HHP and control groups was not statistically significant (*p* = 0.07)

The results of the compressive test are shown in Fig. 6. The maximum compressive load of the untreated control group was 1568.9 ± 459.3 N (coefficient of variation (CV) = 29.3%), whereas those of the HHP-treated and liquid nitrogen-treated groups were 1238.9 ± 177.7 N (CV = 14.3%) and 1047.9 ± 247.8 N (CV = 23.6%), respectively. Both treated groups showed a lower maximum compressive load compared with the control group. A statistically significant difference was observed between the liquid nitrogen-treated and control groups (*p* = 0.003; Cohen’s d = 1.41), whereas the difference between the HHP-treated and control groups was not statistically significant (*p* = 0.07; Cohen’s d = 0.95). No significant difference was observed between the HHP-treated and liquid nitrogen-treated groups (*p* = 0.39).

### Three-Point Bending Test (Maximum Load at Failure)

**Fig. 7:**
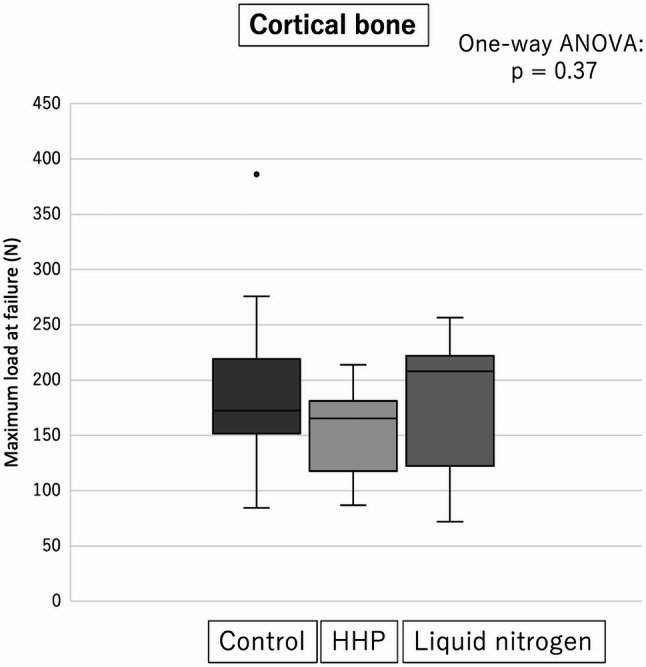
Maximum load at failure of cortical bone after HHP, liquid nitrogen, or no treatment. Legend: Box plots representing the median, interquartile range, and range of the maximum load at failure for beam-shaped cortical bone specimens (*n* = 12 per group). No statistically significant differences were observed among the groups (one-way ANOVA, *p* = 0.37)

Figure 7 shows the results of the three-point bending test. The mean maximum loads at failure were 192.8 ± 78.4 N (CV = 40.7%) for the control group, 155.6 ± 41.9 N (CV = 26.9%) for the HHP-treated group, and 176.5 ± 65.5 N (CV = 37.1%) for the liquid nitrogen-treated group. The maximum loads at failure did not differ significantly among the three groups (one-way ANOVA, *p* = 0.37).

## Discussion

This study evaluated the effects of HHP and liquid nitrogen treatments on the mechanical properties of fresh porcine trabecular and cortical bone. In the compression test using trabecular bone cubes, both treatment groups showed a tendency towards a reduced load at failure compared with the untreated controls; however, only the liquid nitrogen-treated group exhibited a statistically significant decrease. In the three-point bending test using cortical bone, no statistically significant differences in maximum load at failure were observed among the groups. To the best of our knowledge, this study is the first to use fresh, unfrozen bone, as opposed to prior studies that relied on frozen or fixed specimens [22, 29]. This methodological distinction is critical, as it eliminates artifacts associated with prior preservation and ensures that the observed mechanical outcomes reflect the intrinsic effects of the devitalization treatments alone. The direct comparison with liquid nitrogen under the same conditions enhances the clinical relevance and could suggest a mechanical advantage of HHP for devitalized bone grafts.

Previous studies showed that bone strength is preserved at HHP pressures of 250–300 MPa but deteriorates above 600 MPa [22, 29]. The 200 MPa for 10 min used in this study represents a milder condition that did not significantly compromise the mechanical properties in both bone types. These findings align with earlier research, which showed that similar HHP conditions can inactivate tumor and stromal cells [23–26] while preserving the structure. The preservation of structural stability is likely owing to multiple factors. Waletzko-Hellwig et al. [30] reported that type I collagen and other matrix proteins retain their structure following HHP treatment. A histological analysis in a recent animal study by the same group also showed no structural damage following HHP treatment [31], which is consistent with the present mechanical results.

Reports on the biomechanical effects of liquid nitrogen have been variable. Yamamoto et al. [27] investigated bovine metatarsal bone, which resembles the distal femur. They found that the compressive strength was maintained following treatment with a one-cycle liquid nitrogen protocol (20 min in liquid nitrogen, followed by 15 min at room temperature and 15 min in saline). However, their analysis focused on the entire bone, without separating the cortical and trabecular components, making it difficult to assess the effect of cryotreatment on each bone type individually. Similarly, Nik Abdul Adel et al. [28] conducted a four-point bending test using sheep tibiae reconstructed with locking plates following treatment by liquid nitrogen or pasteurization. They reported no significant differences in mechanical strength between the treated groups and untreated controls. However, the mechanical testing was performed following fixation with plates and screws. Consequently, the results likely reflect the properties of the fixation construct, rather than the intrinsic strength of the treated bone itself. Our study avoided fixation and evaluated the cortical and trabecular bones separately, offering a clearer assessment of intrinsic strength. Under these controlled conditions, both devitalization methods showed a trend towards a reduced maximum compressive load at failure in trabecular bone, with the reduction being more pronounced in the liquid nitrogen-treated group, which showed a statistically significant difference compared with the control. This finding suggests a potential disadvantage of liquid nitrogen-treated grafts in applications involving load-bearing trabecular bone.

The differing mechanical outcomes between cortical and trabecular bone can be attributed to their distinct structural characteristics. Cortical bone, with its high density and orderly arranged collagen fibers, exhibits robustness against physical stresses [32]. Its low porosity likely limits the rapid infiltration and expansion effects of freezing agents. In contrast, trabecular bone is structurally more susceptible to processing stresses owing to its high surface-to-volume ratio and porous architecture [33]. The marked reduction in trabecular bone strength following liquid nitrogen treatment may be explained by freeze–thaw-related microdamage affecting this porous network disproportionately. Ice crystal formation within water-rich marrow spaces and the surrounding matrix can generate volumetric expansion and local stresses during freezing and thawing, potentially initiating microcracks and weakening the thin trabecular struts [34]. In addition, freeze–thaw cycles have been shown to destabilize the hierarchical organization of collagen, primarily through ice formation [35]. Changes in matrix hydration are also relevant: water associated with the collagen–mineral matrix contributes to fracture resistance and energy absorption, and altering these water–matrix interactions can reduce bone toughness [36, 37]. Collectively, these mechanisms offer a plausible rationale for why trabecular bone may lose strength, while cortical bone strength is relatively preserved under freezing-based devitalization protocols. Whereas liquid nitrogen treatment caused significant damage, likely owing to ice crystal expansion within this delicate porous network, HHP treatment also showed a trend towards reduced strength. This suggests that the trabecular network is inherently more vulnerable to external physical stressors compared with the robust cortical bone.

Beyond biomechanical outcomes, HHP offers practical advantages in surgical workflows. Liquid nitrogen treatment involves multiple steps, namely immersion, thawing, and warming, which require over 45 min [27]. HHP treatment is completed in a single 10-min cycle and can be easily standardized, potentially reducing the operative time. Although this study focused on mechanical strength, biological outcomes are also important. Liquid nitrogen-treated bone supports bone healing, partly through the formation of a fibrous membrane that is rich in angiogenic activity and growth factor secretion [38]. However, HHP-treated bone has also shown promising osteogenic potential. Recent studies have reported that HHP-treated allografts promote osteogenic differentiation of mesenchymal stem cells and preserve osteoinductive properties, both in vitro and in vivo [39].

One limitation of HHP treatment lies in the size of the available processing chambers. The pressure vessels that are currently in use accommodate specimens up to approximately 50 mm in diameter and 110 mm in height. This capacity is sufficient for preparing the bone chips or small block grafts that are often used in spinal fusion or cavity filling. However, it restricts the treatment of larger anatomical structures, such as whole femurs or tibial shafts. Therefore, expanding the capacity of HHP equipment remains an important technical challenge for future clinical applications. However, the existence of large-scale industrial equipment (e.g., > 300 L capacity) in the food sector [40] suggests significant potential for successfully scaling up this technology.

This study has several limitations. First, only in vitro mechanical testing was performed using fresh porcine bone. Although this allowed for controlled evaluation of the structural effects following treatment, biological responses such as osteointegration and bone healing were not assessed. Further in vivo studies using animal models are required to evaluate revascularization and long-term remodeling under these specific treatment conditions. Second, the sample size (*n* = 10–12) was relatively small. Although a sensitivity analysis suggested sufficient power to detect large differences (e.g., liquid nitrogen vs. control), it may have been insufficient to confirm smaller differences, such as the near-threshold but non-significant reduction observed in the HHP group (*p* = 0.07; Cohen’s d = 0.95). Future studies with larger sample sizes are needed to confirm whether this near-threshold trend is reproducible. Third, simplified specimen shapes and loading conditions were used: cubic trabecular bone under compression and beam-shaped cortical bone under three-point bending. These models do not fully reflect the anatomical complexity and mechanical demands of clinical settings. Future studies should incorporate cyclic fatigue testing and microstructural analysis (e.g., micro-CT) to characterize the long-term mechanical properties fully. Finally, in the HHP protocol, a pressure of 200 MPa was selected based on previous reports of tumor inactivation. Future research should explore a wider pressure range (e.g., 300–600 MPa) to identify the optimal therapeutic window that maximizes devitalization efficiency while maintaining mechanical integrity.

## Conclusions

Although liquid nitrogen treatment significantly reduced the load at failure of trabecular bone compared with the untreated control, the reduction following HHP treatment did not reach statistical significance. In the three-point bending test of cortical bone, no statistically significant differences in maximum load at failure were observed among the groups. These findings provide biomechanical evidence to support the use of HHP for preparing devitalized bone grafts, particularly for load-bearing applications.

## Data Availability

The datasets generated and analyzed during the current study are available from the corresponding author upon reasonable request.

## Abbreviations

ANOVA: Analysis of variance
CV: Coefficient of variation
HHP: High hydrostatic pressure
SD: Standard deviation
